# Evolving Philosophies of Alignment in TKA: From Mechanical Uniformity to Personalised Harmony

**DOI:** 10.3390/medicina62020307

**Published:** 2026-02-02

**Authors:** Hong Yeol Yang, Jong-Keun Seon, Khairul Anwar Ayob

**Affiliations:** 1 Department of Orthopaedic Surgery (Knee & Sports Medicine), Chonnam National University College of Medicine, Hwasun 58128, Republic of Korea; stephano.h.yang@gmail.com; 2 National Orthopaedic Centre of Excellence for Research and Learning, Department of Orthopaedic Surgery, Faculty of Medicine, University Malaya, Kuala Lumpur 50603, Malaysia

**Keywords:** total knee arthroplasty, alignment philosophy, kinematic alignment, functional alignment, robotic-assisted knee arthroplasty

## Abstract

*Background and Objectives*: Mechanical alignment (MA) has long been the gold standard in total knee arthroplasty (TKA), aiming for neutral hip–knee–ankle alignment with proven long-term survivorship. However, up to 20% of patients remain dissatisfied, often due to neglect of individual constitutional limb variation and subsequent soft tissue imbalance. This has driven the development of alternative alignment philosophies. This current concepts review aims to determine the various evolving alignment strategies, elucidate their underlying principles, and demonstrate the available clinical outcomes data. *Materials and Methods*: This review examines MA and the paradigm shift towards personalized alignment techniques, including Kinematic Alignment (KA), restricted Kinematic Alignment (rKA), inverse Kinematic Alignment (iKA), adjusted mechanical alignment (aMA), and the most recent evolution, Functional Alignment (FA). *Results*: Kinematic alignment and its derivatives (rKA, iKA) seek to better replicate native joint morphology and tension, often reducing the need for soft tissue releases and improving functional outcomes compared to MA. rKA and iKA introduce protective boundaries to avoid extreme phenotypes and possible instability. FA leverages robotic platforms and integrates these principles with real-time gap balancing, demonstrating promise for consistent, personalized outcomes. Some reports, however, advise caution with adjusted Mechanical Alignment (aMA), particularly those that result in phenotypes such as Coronal Plane Alignment of the Knee (CPAK) VII or VIII, which may increase the risk of revision. *Conclusions*: The philosophy of TKA has evolved from a uniform mechanical target (MA) to a more nuanced, patient-specific strategy. While promising mid- to long-term outcomes and comparable survival data support the viability of KA and its derivatives, critical needs remain, including standardizing nomenclature (especially for FA) and conducting high-quality comparative trials. Future directions involve leveraging high-volume intraoperative data and Artificial Intelligence (AI) to refine decision-making and further personalize alignment strategies, without compromising long-term implant survivorship.

## 1. Introduction

The optimal implant orientation in total knee arthroplasty (TKA) remains a subject of ongoing debate. Historically, restoration of a neutral mechanical axis has been considered essential for optimizing postoperative outcomes after TKA [1,2]. Mechanical alignment (MA), characterized by achieving a hip–knee–ankle (HKA) angle of 0°, has served as the prevailing paradigm [2,3,4] based on the principle that neutral limb alignment distributes load symmetrically across the tibiofemoral compartments, thereby mitigating polyethylene wear and minimizing the risk of aseptic loosening [5].

**Figure 1:**
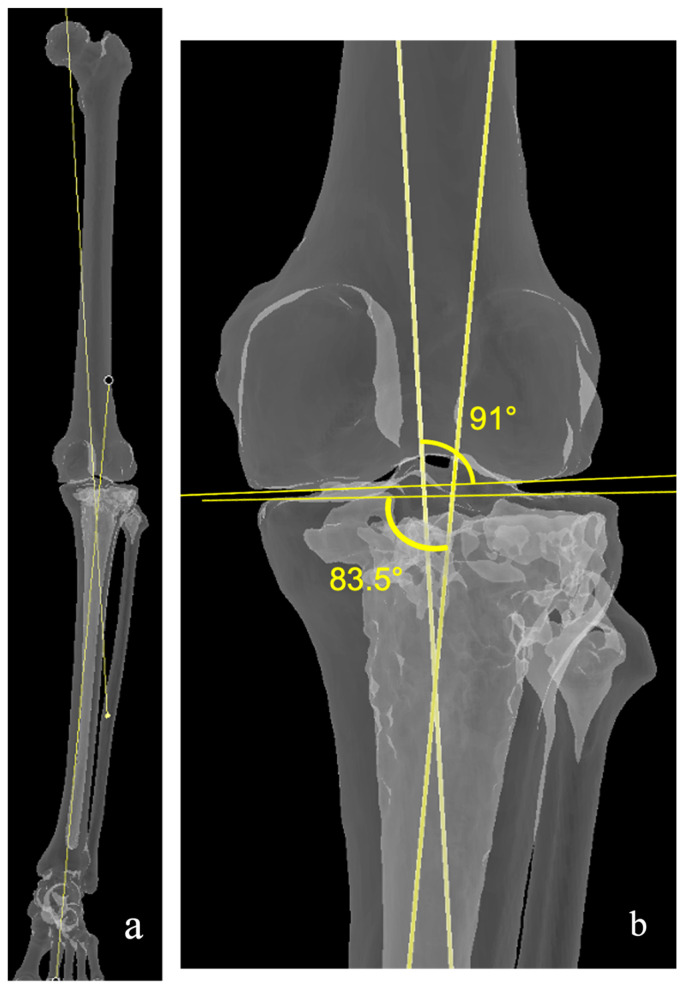
Typical CPAK I knee, which will be used to demonstrate the different alignment philosophies. (**a**) Long view of the limb with mechanical axes of the femur and tibia marked (**b**) Close-up with anatomical measurements. Medial Proximal Tibial Angle (MPTA): 83.5°, Lateral Distal Femoral Angle (LDFA): 91°. HKA: −7.5°. JLO: 174.5°, Apex distal.

Although MA in TKA has yielded generally satisfactory results, up to 20% of patients remain dissatisfied postoperatively [6,7,8], with discomfort during routine activities cited as a prominent contributing factor [9,10,11]. A pivotal study by Bellemans et al. [12] initiated substantial discourse regarding the optimal alignment target. They demonstrated that constitutional limb alignment fell outside the 0° ± 3° range in 34% of males and 20% of females within a non-arthritic population. Hirschmann et al. [13,14,15] introduced a more recent classification system, incorporating both femoral and tibial mechanical alignment angles (FMA and TMA) to better characterize individual coronal plane alignment phenotypes. Figure 1 represents a typical arthritic knee with a varus joint line.

Consequently, imposing a neutral mechanical axis may deviate from a patient’s native limb alignment, potentially inducing soft-tissue imbalance and kinematic conflicts. The optimal alignment strategy for maximizing patient-reported outcome measures (PROMs) remains contentious, prompting the development of alternative alignment philosophies that aim to replicate the individual’s pre-arthritic alignment (Figure 1). These emerging techniques reflect a paradigm shift toward more personalized approaches in TKA [4,16,17,18,19,20,21]. However, the principles underpinning these philosophies, the boundaries they employ, and the robustness of their clinical outcomes remain varied and heterogeneous.

The objective of this current concepts review is to critically evaluate contemporary alignment strategies in TKA, elucidate their underlying principles, and present available clinical outcomes data, with particular emphasis on PROMs, alignment boundaries, and implant survivorship.

This study is a narrative current concepts review conducted using a structured literature review approach. Literature identification focused on concept-defining and clinically influential studies across alignment philosophies, supplemented by a targeted PubMed search for contemporary evidence. A formal systematic review or meta-analysis was not performed due to heterogeneity of study designs and outcomes.

## 2. Legacy Alignment Philosophies

### 2.1. Mechanical Alignment

**Figure 2:**
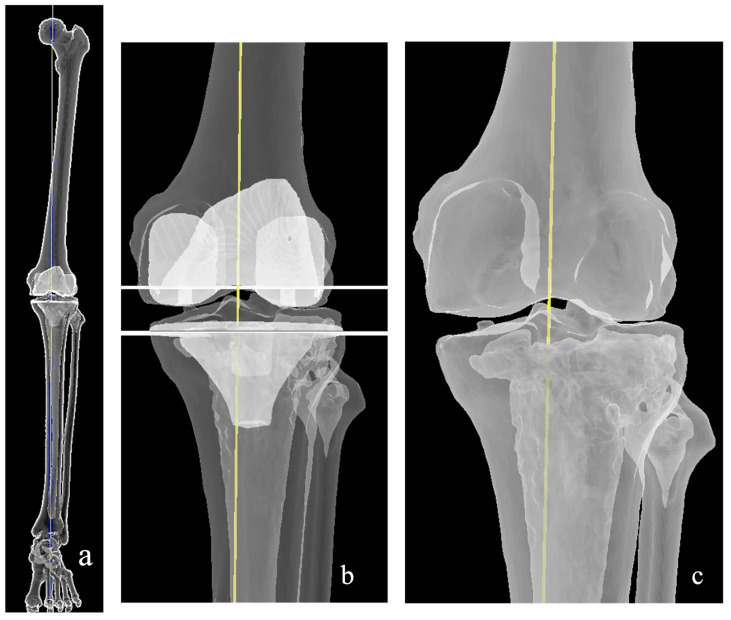
Mechanical alignment: (**a**) components positioned perpendicular to the resultant axis. (**b**) Both components are aligned to the mechanical axis, resulting in less overall bone removed medially (**c**) Note the larger gap at the medial side once the tibia is aligned to the femur, indicating the need for more medial side release.

The MA technique aligns both the femoral and tibial components perpendicular to their respective mechanical axes, thereby achieving a neutral HKA (Figure 2). Since its introduction by Insall et al. [22] nearly four decades ago, MA has remained the predominant standard for TKA in the world. The underlying rationale is to optimize load distribution across the tibiofemoral compartments and enhance implant longevity. In this approach, coronal alignment is achieved by performing bone resections perpendicular to the mechanical axes of the distal femur and proximal tibia. Sagittal alignment is dictated by individual patient anatomy and is influenced by prosthesis design and the degree of constraint used. Rotational alignment of the femur can be determined using either the gap-balancing technique, which references soft-tissue tension, or the measured resection method, which primarily relies on anatomy [23,24].

Although MA was developed with the intent of maximizing implant survivorship, it purposely overlooks individual variation in lower limb alignment, morphology, and native biomechanics. Consequently, it may lead to complications, including increased patella-femoral retinacular tension and lateral column lengthening due to distal femoral component overstuffing [25]. Furthermore, by ignoring the native joint line height and obliquity, MA can lead to knee imbalance that may be technically uncorrectable [26].

MA has long been regarded as the gold standard in TKA, with multiple studies reporting favorable clinical outcomes and implant survivorship rates ranging from 89% to 99% at 10 years, and 85% to 97% at 20 years of follow-up [27,28,29]. Early evidence in favor of MA derived from previous reports that are associated with deviations from neutral alignment, particularly tibial component mal-positioning, to increased failure rates [1,2,5,30]. However, these findings were derived from procedures utilizing old prosthesis designs, non–cross-linked polyethylene inserts, and manual instrumentation with a lack of precision. Moreover, alignment assessment in these studies was often based on short-leg radiographs rather than full-length weight-bearing radiographs (FLWBR). In a 15-year follow-up study of 501 TKAs using Press Fit Condylar cruciate-retaining prostheses, Bonner et al. [16] demonstrated a 10-year revision rate for aseptic loosening of 5% in knees within ±3° of neutral mechanical axis versus 14% in those outside this range, as determined by FLWBR. While registry data showing 82% survivorship at 25 years continues to support the durability of MA, more recent investigations utilizing FLWBR have reported no significant difference in long-term implant survivorship that are positioned outside the traditional ±3° MA target [21,31,32].

The strength of evidence supporting mechanical alignment lies mainly in its extensive history, along with its large numbers from studies and registries. These data provide robust reassurance in regard to its durability. However, much of this evidence originates from earlier generations of implant designs and more conventional instruments, limiting its direct extrapolation to current practices. The introduction of modern prosthesis designs and advanced instrumentation, along with comparable survivorship rates and a paucity of well-controlled comparative studies, has intensified the ongoing debate regarding the universal applicability of MA in TKA.

### 2.2. Anatomical Alignment

**Figure 3:**
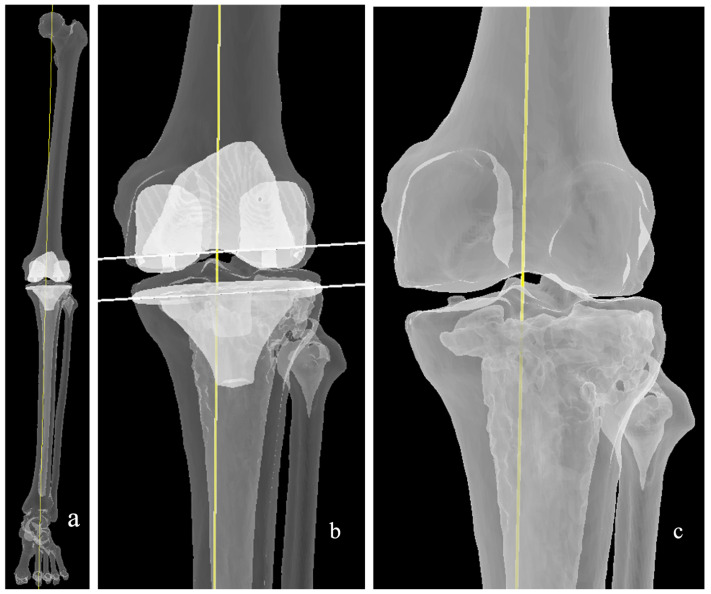
Anatomical alignment (**a**) target is for a straight limb alignment (**b**) systematic positioning of components 87 degrees to the mechanical axis. (**c**) Similar to MA, a larger gap on the medial side indicates more bone removal from the medial side.

Anatomical alignment (AA) was first proposed by Hungerford and Krackow in the 1980s with the intention of improving knee function by more closely replicating native alignment [23]. This technique involves a tibial resection in 3° of mechanical varus and a distal femoral resection in 3° of mechanical valgus, aligning the femoral component with the posterior condylar axis (PCA) (Figure 3). Advocates of AA suggest it facilitates better load distribution across the tibial component and enhances patellofemoral biomechanics by minimizing ligament strain during flexion [33]. However, limitations in prosthesis design at the time, particularly with the Porous-Coated Anatomic prosthesis, and the technical difficulty of obtaining accurate bone resections without inducing excessive varus alignment (>3°) hindered its widespread adoption [23]. AA is now largely considered a conceptual precursor to kinematic alignment.

In a randomized controlled trial, Yim et al. [34] compared AA and MA in robotic-assisted TKA using a cruciate-retaining prosthesis and found no significant differences between the groups in terms of range of motion, functional scores, and joint laxity at two-year follow-up. Similarly, Yeo et al. [35] evaluated AA versus MA with robotic-assisted TKA over a mean follow-up of eight years and reported no meaningful differences in joint stability, functional outcomes, and gait analysis.

Evidence supporting AA is limited by relatively small sample sizes and a paucity of long-term follow-up. The available evidence demonstrates equivalence to MA in terms of function and survivorship, although these reports are underpowered to detect subtle differences in satisfaction. As such, AA is best regarded as a conceptual bridge between MA and kinematic philosophies rather than a fully validated standalone strategy.

### 2.3. Kinematic Alignment

Kinematic alignment (KA), introduced by Howell et al. in 2006, is a patient-specific technique designed to restore the native, pre-arthritic alignment of the limb and joint line [36,37,38,39]. This method conceptualizes the knee around three kinematic axes in relation to the posterior and distal femoral joint lines: a femoral transverse axis for tibiofemoral flexion-extension, a second axis about which the patella extends and flexes, and about which the tibia externally and internally rotates on the femur. These three axes are oriented parallel or perpendicular to the native joint lines [40]. KA involves resurfacing the femorotibial joint to align implant components with these kinematic axes and joint lines of the native joint. The femoral component is positioned to preserve the native joint line obliquity, while the tibial resection is tailored to balance flexion and extension gaps. Restoration of native femoral anatomy is prioritized, with tibial recuts or selective soft-tissue releases performed for cases in which acceptable ligament balance cannot be achieved.

**Figure 4:**
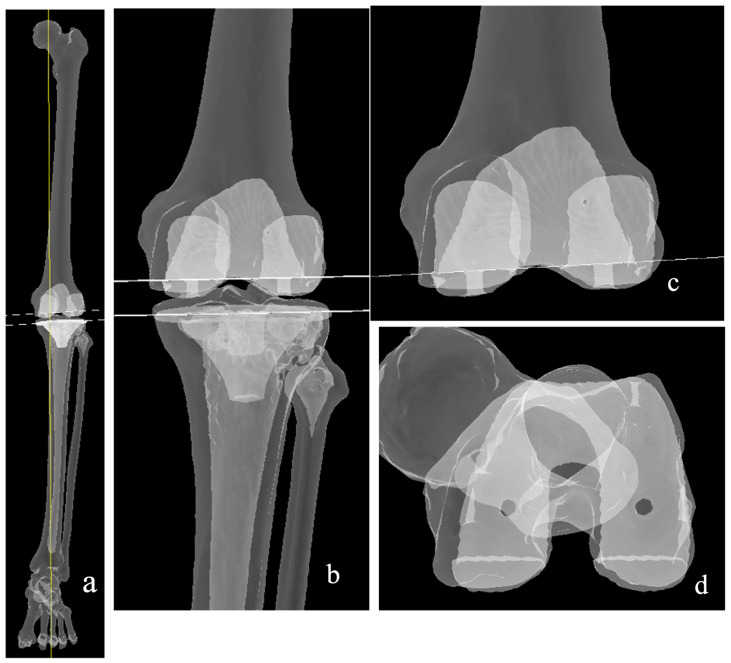
Kinematic alignment: (**a**) The resultant coronal alignment (yellow line) matches the native HKA angle. (**b**,**c**) Resurfacing of the femur is performed, with considerations for wear and kerf of the saw blade. Cut surfaces marked with the white lines. (**d**) Medial and lateral posterior femoral cuts are usually performed symmetrically, i.e., parallel to PCA, assuming no wear.

In KA, the thickness of femoral and tibial resections is verified using calipers to match the corresponding implant dimensions, accounting for cartilage wear and saw blade kerf (Figure 4). This approach aims to preserve native ligament laxity and maintain balanced flexion and extension gaps, thereby reducing the need for soft-tissue releases [41]. Howell’s original protocol does not allow any restrictions on native anatomy or postoperative alignment correction. Execution of KA demands a high degree of surgical precision and can be performed using conventional instrumentation, computer navigation, patient-specific instrumentation (PSI), or robotic assistance, with caliper verification ensuring the accuracy of resections [41,42].

By preserving native anatomy, KA has been associated with improved functional and clinical outcomes compared to alignment techniques that drive the limb into an unnatural position [43,44,45]. A principal drawback with unrestricted KA, however, lies in the potential to replicate extreme anatomical alignments, particularly when using patient-specific cutting guides (PSGs) or conventional instrumentation, which may lack the precision to avoid malaligned outliers. Despite these concerns, multiple short- and mid-term studies have demonstrated that reproducing constitutional varus or valgus alignment, femorotibial joint line obliquity, or tibial plateau varus inclination does not adversely affect implant survivorship or clinical outcomes [17,38,42,46,47,48,49]. Supporting this, a registry analysis from Australia and New Zealand reported comparable seven-year revision rates between TKAs performed with unrestricted KA using PSGs (3.1%) and those performed with conventional or computer-assisted techniques (3.0%) using the same posterior cruciate-retaining implant. Recent long-term data further support the durability of KA. In a 10-year follow-up study, Howell et al. [38] demonstrated excellent functional outcomes and implant survivorship, with no increase in tibial component loosening, even in patients with constitutional varus alignment. Building on these findings, a subsequent 16-year follow-up of 222 TKAs performed with unrestricted KA reported a 93% survivorship and a 7% reoperation rate, outcomes that are comparable to or better than those historically reported for MA [50].

Kinematic alignment is supported by a growing body of prospective, randomized, and registry-based evidence demonstrating improved PROMs and non-inferior survivorship, at least in the medium term. Strengths of this evidence include caliper-verified execution, phenotype-driven rationale, and long-term follow-up of up to 16 years. However, concerns remain regarding reproducibility and the risk of extreme alignment outliers, particularly when executed without navigation or robotic assistance. Consequently, while the evidence base for kinematic alignment is among the strongest of the alternative philosophies, its safe application is highly dependent on surgical accuracy and experience.

## 3. Emergence of Alternative Alignment Philosophies

As the techniques in performing knee arthroplasties evolve, specifically with the use of navigation, which has now evolved to robotic-arm assisted surgeries, and also to a lesser extent PSI, the level of accuracy and consistency offered allowed surgeons to re-define their targets, balancing between prosthesis longevity and the patient’s subjective perception on the “naturalness” of the knee [51,52,53,54,55,56].

The advent of optical navigation being one factor, a more robust polyethylene being another [57], the aims of a modern TKA are now less concerned with inadvertent deviations in alignment; instead, it is more concerned with a balanced gap medially and laterally, with some allowances given to the lateral flexion space. As the accuracy of the resultant bone cuts is achieved at the sub-millimeter level [58], the worry of shear stresses to the polyethylene and bone cement is reduced, and more attention can be given to aim for restoration of the pre-morbid collateral ligament tension and knee phenotype.

That said, the availability of robotic-assisted platforms remains relatively limited, and each platform has its own advantages and disadvantages. Descriptions of performing an alternative alignment philosophy in TKAs using conventional instrumentation have been reported, although these techniques carry the usual downsides of conventional TKAs [59,60].

### 3.1. Restricted Kinematic Alignment

**Figure 5:**
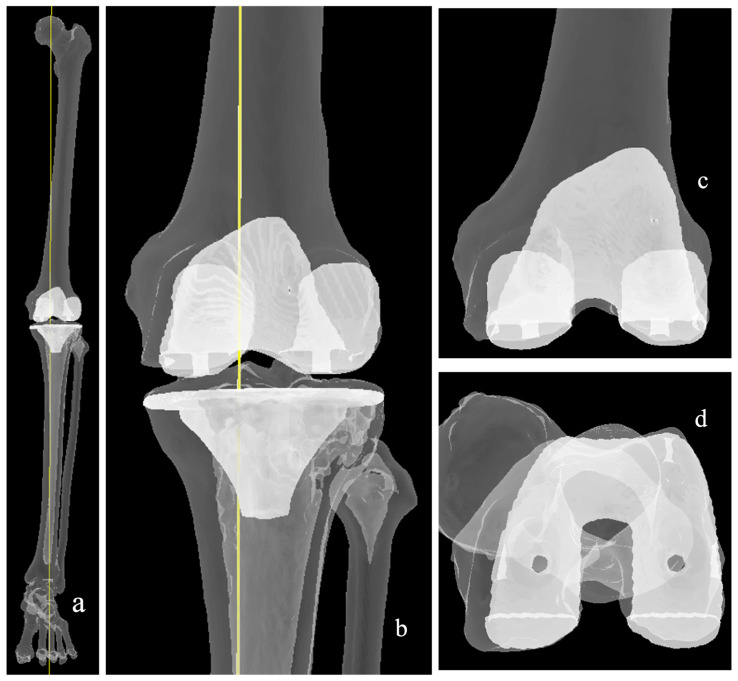
Restricted kinematic alignment (rKA). (**a**) Boundaries are followed in this case, the tibia orientation is restricted to 5 degrees to its mechanical axis. (**b**,**c**) The resultant alignment or hip knee angle (yellow line) is less than 3 degrees. These adjustments may also alter the rotation of the femoral component (**d**) to achieve a balanced gap.

The initial description of the restricted kinematic alignment (rKA) philosophy was by Vendittoli, from Montreal, Canada, who modified the kinematic alignment philosophy in 2011 [61]. The aim, similar to the original objective of Dr Stephen Howell, is to resurface the knee, restore the native joint surface orientation, and pre-morbid alignment. Dr Vendittoli was, however, concerned about the high variability in knee morphology and sought to address and prevent outliers in his patient population (Figure 5). The five principles of the rKA are as follows:

1. arithmetic HKA angle should be ±3°
2. femoral and tibial joint orientations should be ±5° from their mechanical axis
3. native joint laxities should be restored (no gap balancing)
4. femoral anatomy preservation should be prioritized
5. anatomical modifications should be performed on the diseased compartment/intact compartment should be resurfaced

The use of calipers for verification of cut depths was possible in 51% of cases; however, among those whose knee morphology deviates from the rKA boundaries, Venditolli has proposed the use of PSI, operative navigation, or robotic-assisted platforms to achieve the principles of rKA. Even in these cases, a caliper can be performed if there are errors in verifying cut surfaces or if collateral laxity falls outside the expected range [62].

The argument for rKA is that, when knees fall outside the “normal” anatomy, the morphology should be considered pathological, and hence it is unwise to blindly recreate the pathological features [63]. In addition, total knee arthroplasty remains a legacy design—largely unchanged since the inception of the total condylar prosthesis (TCP)—with only a few minor iterations from different prosthesis manufacturers and newer designs. These prostheses may not be able to withstand the resultant loads if the prosthetic knee components are placed in outlier anatomy [5].

The principles of the rKA aim to serve as a safety net while respecting most of the suggestions in the original KA description. Knees that fall outside of the suggested parameters may require some ligamentous release to achieve mediolateral balance in flexion and extension. As mentioned, further tuning the HKA and joint orientation, as well as achieving the desired collateral balance, may require, at the very least, computer navigation.

In comparison to MA, the rKA philosophy reduces the need to alter the pre-existing joint angles, suggesting less of a change to the normal knee biomechanics [64]. In full extension, the collateral laxities are also more consistently balanced with the rKA [65].

Clinically, patients who underwent knee arthroplasty with the rKA concept have satisfactory functional outcomes and noninferior survivorship at the 10-year mark [66]. Even in the hands of independent surgeons, rKA has been shown to produce, at the very least, equal improvement in patient-related outcome measures and satisfaction [67,68].

The principal strength of evidence for rKA lies in its pragmatic balance between personalisation and safety. Prospective cohort studies demonstrate satisfactory functional outcomes and mid-term survivorship, while clearly defined boundaries mitigate concerns about leaving the patient at the extremes of alignment. However, long-term data beyond 10 years remain limited, and most studies originate from high-volume centers with access to advanced technologies. As such, rKA currently represents a clinically appealing compromise, supported by moderate-quality evidence.

### 3.2. Inverse Kinematic Alignment

**Figure 6:**
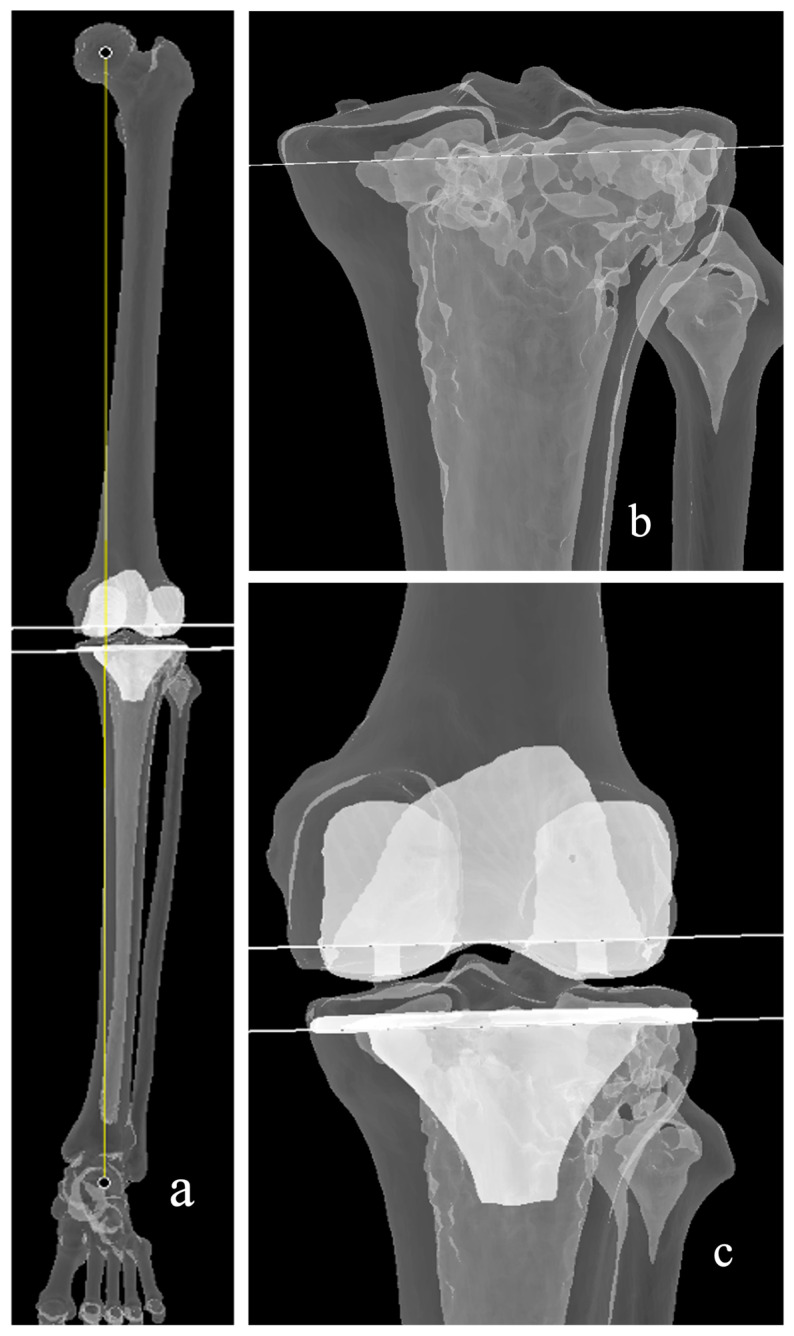
Inverse kinematic alignment, where the resurfacing is considered mainly at the tibia (**b**), (**c**); i.e., equal bone cuts (while lines) of both tibial plateaus. Subsequent femoral cuts are balanced accordingly. (**a**) The resultant alignment matches the native alignment (yellow line).

The inverse kinematic alignment (iKA concept) was first described by Winnock de Grave [69], altering the paradigm from using the femur as the benchmark, and subsequently balancing the tibial cut to the ligament tension in KA, to using the tibia as the initial reference site (Figure 6). The tibial plateau is cut equally at the medial and lateral compartments, taking the anterior two-thirds and posterior third as the reference point. The original description considers only the bone to determine the cut height, as the cartilage is likely worn. This is most suitably performed via a CT-based preoperative planning, which is the system used when this philosophy was first described. However, since the first description, a number of authors have described the use of other platforms to achieve the same philosophy, with similarly good results [70,71].

Once the tibia cut has been performed, the femur cuts’ position in the sagittal, coronal, and axial planes is then decided upon based on the tension of the collateral ligaments in extension and flexion. The sagittal position of the tibia is dependent on the prosthesis of choice. A cruciate-retaining implant will be matched to the native posterior slope, whereas in cruciate-sacrificing implants, the slope is reduced.

Similar to rKA, this philosophy applies boundaries to the cut dimensions, specifically restricting the Medial Proximal Tibial Angle (MPTA) to 84–92, the mechanical Lateral Distal Femoral Angle (mLDFA) to 84–93, and the HKA to 174–183 [69]. Similar to the restricted kinematic principle, accuracy is of utmost importance to ensure minimal to no deviation from the boundaries; hence, optical navigation and robotic arm execution are used. There have, however, been descriptions of the use of iKA philosophy with conventional instruments [59,60].

This proposed concept reduces the amount of varus created by neutralizing the varus formed by the cartilage (i.e., the JCLA), which, on average, is 3 degrees [72]. When compared with the adjusted mechanical alignment (aMA) and rKA, this philosophy accommodates a wider range of native knee phenotypes, with only about a quarter requiring adjustments to the MPTA and even fewer to the LDFA [73].

Compared with the aMA, at the 12-month mark, a greater proportion of patients with iKA were satisfied. Both methods used the tibia as the starting point, equal bony resections in the iKA, within the allowed safe zones, while the aMA group started with the tibia at 90 degrees to the tibial axis. The femoral cuts were then adjusted to achieve a laxity of 1–2 mm in both compartments [74].

This philosophy is supported predominantly by short- to medium-term cohort studies. While early data suggest improved PROMs, the absence of long-term survivorship data, along with reliance on technology-intensive execution, may constrain broader adoption. The current evidence base should therefore be considered hypothesis-generative rather than definitive.

### 3.3. Adjusted Mechanical Alignment

**Figure 7:**
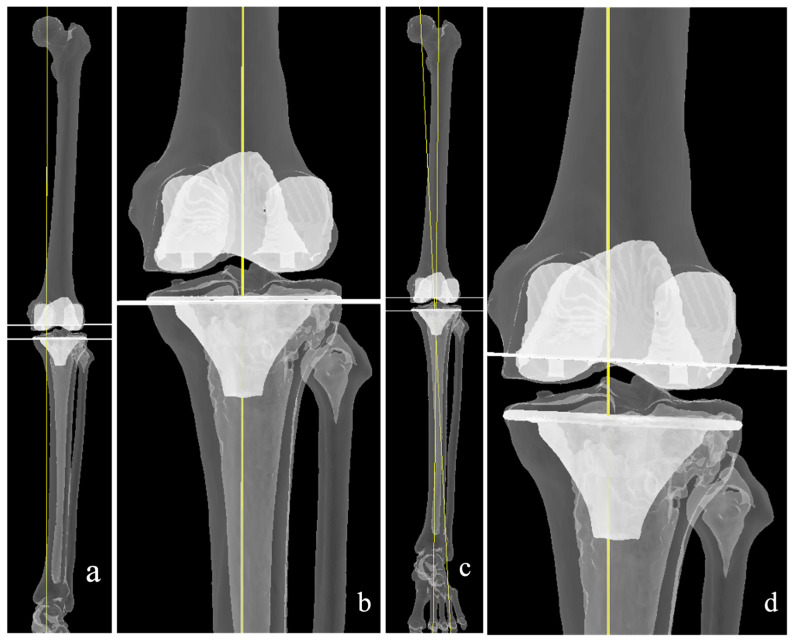
(**a**) Adjusted mechanical alignment. In this philosophy, the tibia is fixed perpendicular to the mechanical axis (yellow line) (**b**,**d**), and the femoral cuts (white line) is adjusted according to the soft-tissue laxities; in this example, the resultant femoral orientation is varus (**c**).

This philosophy stems from the principle of mechanical alignment, i.e., to obtain the alignment as perpendicular to the mechanical axis as possible. At the same time, the position of the components was adjusted to respect the soft tissue as much as possible (Figure 7).

The first description of the adjusted mechanical alignment was by Vanlommel et al. [20], where they retrospectively observed that patients with preoperative varus alignment scored better PROMs when left in mild varus (>177 and <174) compared to those who were corrected to neutral (180 ± 3) or left in severe varus (>174). Their recommendation is that mild undercorrection of a varus deformity is desired; however, it is not advisable to go beyond 6 degrees. However, the authors did not specify the location from which the varus was arising, either the femur or the tibia.

This philosophy is then adopted as a way to be true to the mechanical alignment, but at the same time limit unnecessary soft tissue releases. Although popular, there is a limited number of academic articles describing how to apply this method. Zheng et al. [75] retrospectively compared MA and aMA. The start point was the tibia, which was always performed perpendicular to the tibial mechanical axis, and the femur coronal position was adjusted to allow for a relatively balanced extension gap (lateral 1–2 mm more laxity). Patients in the aMA group were described to have better functional outcomes at a mean of 7.2 years. However, this was not statistically significant. They noted that patients in the aMA group had higher HSS scores at the 1-month mark, and this was statistically significant.

Lee et al. reported a significant association between aseptic loosening of the tibial component and postoperative HKA angles exceeding 3 degrees from neutral alignment [3]. The incidence of loosening was found to increase progressively with greater deviation from neutral HKA. Upon further stratification, a substantial proportion of the malaligned cases exhibited increased varus alignment originating primarily from the femur rather than the tibia. Interestingly, when the femur was aligned closer to neutral, the correlation with tibial component loosening was markedly reduced, suggesting that femoral malalignment may play a more critical role than previously appreciated. A similar finding was also reported by Yang et al., who found that TKAs outside of 3 degrees to the mechanical axis and with a lateral joint line inclination were associated with the highest aseptic loosening rate at 12 years [76]. This finding may challenge the aMA philosophy, where a high number of surgeons describe starting with the tibia in neutral, and adjusting the femur according to the soft tissue tension.

The evidence for aMA is largely retrospective and heterogeneous in execution. While several studies suggest improved early functional outcomes with nomenclature compared to strict mechanical alignment, the literature is inconsistent in its use of nomenclature. Emerging data links femoral malalignment to impaired survivorship, which should be further explored with more high-quality studies to validate this hypothesis. At this point, the strength of the evidence for or against aMA remains limited and context-dependent.

### 3.4. Functional Alignment

This philosophy is currently the prevailing approach when performing robotic-assisted knee arthroplasty. First described by Clark in 2022, this alignment principle balances the soft-tissue condition of the knee with the preoperative knee morphology [77].

The attractiveness of this philosophy lies in its alignment with the principles of rKA, iKA, and AMA. The functional alignment philosophy is versatile and builds on the surgeon’s initial alignment philosophy, whether kinematic, inverse kinematic, or adjusted mechanical. The starting point is determined by the surgeon, and the subsequent steps can then be determined based on the desired soft tissue tension or component position, guided by the inputs from the robotic platform.

**Figure 8:**
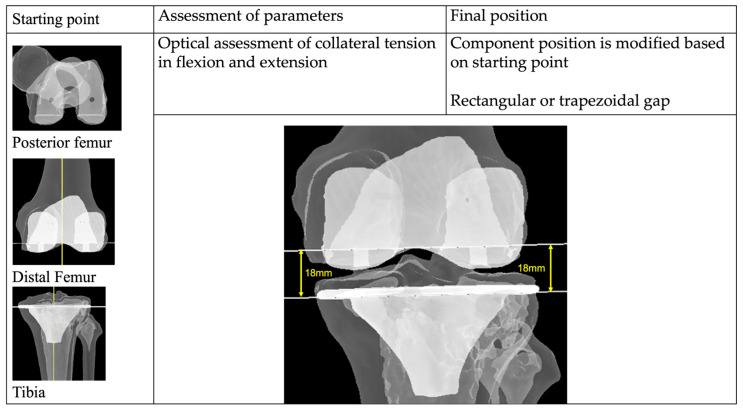
Functional alignment is a versatile philosophy that builds on the surgeon’s initial alignment philosophy. The starting point is determined by the surgeon, and adjustments are made to the surgeon’s final target. Mechanical alignment of the femur and tibia (yellow lines), cut surfaces (white lines).

In the initial description of the FA, Clark described having two starting points, either KA or MA. The KA group started with equal bony resections on the distal and posterior femurs, based on the preoperative CT scans [78]. The position of the femur and tibia is then adjusted according to the mediolateral soft tissue tensions in flexion and extension (Figure 8). Ultimately, the aim of this philosophy is to achieve the primary goal of relatively equal collateral tension in flexion and extension, through minimal or no soft tissue releases. It is interesting to note that in an exceedingly high number of knees, the final tibia alignment is right at the boundary of 6 degrees of varus, although the exact number was not reported.

The original description applied boundaries to the prosthetic position and the final alignment result; adjustments and releases were performed if it ended up outside the recommended parameters. Since its introduction, a large number of publications have reported the consistency of the balance achieved, as well as improved short- to medium-term patient experience with this concept [79,80,81,82]. In a recent randomized controlled study by Young et al., sub-analysis of different CPAK groups revealed that patients with a constitutionally varus knee (CPAK type I) reported higher Forgotten Joint Score (FJS) and Knee Injury and Osteoarthritis Outcome Score, Quality of Life (KOOS QOL) scores with a functionally aligned TKA [81].

It goes without saying that the ability to execute the functional alignment philosophy is highly dependent on recent advances in arthroplasty technology, especially optical navigation, which objectively provides the surgeon with a gap for the prosthesis to fill and tension out. The precision of these optical trackers is submillimeter-level and a game-changer for surgeons’ ability to assess gaps. It is now becoming clearer that robotic-assisted platforms help surgeons ultimately achieve better functional results and patient satisfaction in knee arthroplasty.

There is, however, a need to standardize the nomenclature surrounding the functional alignment philosophy. In a study by Klasan et al. [83], it was shown that, on average, 3.5 different strategies are used to achieve a functional alignment philosophy for 10 phenotypically different knees. The different methods yielded distinct consequent femoral, tibial, and collateral laxity profiles. In the interest of enhanced clarity and reproducibility, it is recommended to subclassify functional alignment according to the starting philosophy (MA, KA, iKA), the component position fixed, and whether a rectangular or trapezoidal space was achieved.

Functional alignment is supported by an expanding body of randomized and prospective studies demonstrating improved early PROMs and satisfaction. Its principal strengths lie in its adaptability and in the integration of real-time soft-tissue assessment. However, most available data remains short- to medium-term, and variability exists in its principles and execution across surgeons. Standardization of terminology and technique is essential to fully isolate the philosophy and its effect on the patient experience before definitive conclusions regarding long-term survivorship can be drawn. Table 1 reviews the differences in the available evidence on each of the alignment philosophies, while Table 2 outlines the recommendations during the execution of the alternative alignment philosophies. 

## 4. Future Directions

**Figure 9:**
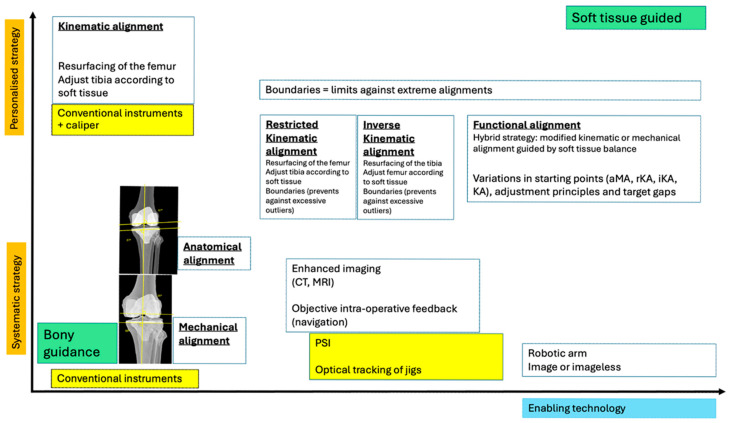
Evolution of the alignment philosophies in TKA. The vertical axis represents the transition from systematic, bone-referenced strategies (MA and AA) towards a more personalized, soft-tissue-respecting strategy (KA, rKA, iKA, and FA). The horizontal axis represents the emergence of enabling technologies, from the early days of conventional instrumentation, including the use of a caliper to validate the thickness of bone removed, the adoption of enhanced imaging modalities to pre-plan and fashion patient-specific instrumentation, and optical tracking for navigation. Finally, the emergence of robotic arms allows for more accurate assessments and executions. Boundaries are present in many alternative alignments to serve as safeguards against extreme alignments and pathological outliers, protecting implant longevity and soft tissue balance.

The evolution of knee arthroplasty has been marked by successive technological and conceptual advances. These include the success of the Total Condylar Knee design, the introduction of modular tibial inserts, and the development of highly cross-linked polyethylene, each contributing to improved implant survivorship and functional outcomes [57,84], as well as the integration of intraoperative navigation systems. Most recently, the development of robotic-assisted platforms [71,85,86,87,88] is considered the most recent revolution. Not only does it allow real-time assessment and feedback on the operative condition of the knee, but it also enables systematic collection of intraoperative data that can be retrospectively analyzed and compared with the patient’s perception of the replaced knee (Figure 9). It is, however, by no means the finished product.

At the moment, even with the ability to visualize or objectify the gaps, there is still significant variability in how to tension the collaterals intraoperatively, as well as in the balance or gaps of choice. Variability in the methods of tensioning the collaterals may under- or overestimate the gaps; hence, the subjective feeling of the knee may differ. Tensor machines have been utilized to provide uniform and equal amounts of tension in every case [89,90]. However, the ideal magnitude of tension, or whether a fixed tension is required for every patient, has yet to be determined [91]. Digital tensioners, although their widespread adoption and impact on surgical workflow have yet to be validated through high-level evidence.

The desired final gap also remains highly surgeon-dependent and is arrived at based on surgical instinct and anecdotal recollection of how the knee and the patient feel [92]. Determining the ideal final tension would be achieved once the critical mass of robotic arthroplasties has been achieved. The aggregation of large-scale intraoperative data will enable correlation between specific gap targets and long-term outcomes, potentially leading to more evidence-based intraoperative decision-making [93,94].

Another frontier lies in personalized alignment philosophies; alterations in the decision to align can also be made based on different patient profiles. Marrying ligamentous tension to alignment philosophies has worked very well for the majority of patients. However, there remains a smaller subset for whom outcomes may vary with conventional philosophies. Patients with less commonly occurring knee phenotypes, morbidly obese patients, amongst other factors, might be less suitable for certain philosophies, and as the field advances, intraoperative decisions may increasingly be guided by data on surgical outcomes, facilitated by artificial intelligence (AI)-driven recommendations from large datasets.

The patellofemoral compartment is an important consideration that has been commonly forgotten or underemphasized in the use of robotic platforms. This is despite the fact that patellar issues cause high dissatisfaction rates and are a significant cause of TKA revisions.

The technology enabling tracking of the patella has not yet matured enough, but once it does, the consideration for resurfacing, the need to adjust prosthesis positioning, and the need for releases should be obtained from the robotic interface [95,96].

Finally, the principles of personalisation and precision in primary TKA should also be extended to patients undergoing revision arthroplasty, especially in the light of the rising revision burden associated with aging populations. This may necessitate a rethinking of revision implant designs, as the position of the prosthesis should not remain constrained to the diaphysis or shape of the metaphysis. Alteration of the prosthesis should now include consideration of the native anatomy and biomechanics, even in the revision scenario.

## 5. Limitations

As a structured narrative review, this study does not provide a systematically assessed synthesis of all available literature, and some degree of selection bias cannot be excluded. Accordingly, the conclusions should be interpreted as a conceptual framework for comparing different philosophies rather than as definitive quantitative evidence.

Several additional limitations warrant consideration. First, the evidence base across alignment philosophies is heterogeneous, encompassing randomized controlled trials, retrospective cohort studies, registry analyses, and conceptual or biomechanical reports, which precludes direct quantitative comparison or meta-analysis. Variations in study design, outcomes, follow-up, and alignment targets further limit cross-alignment comparability.

Second, much of the supporting data for the newer alignment philosophies—particularly rKA, iKA, and FA—are derived from short- to medium-term follow-up and frequently from high-volume centers or designer surgeons with access to advanced technologies. These factors may limit generalizability to lower-volume settings or to environments without routine access to navigation or robotic-assisted platforms.

Third, PROMs, while increasingly emphasized, are subject to ceiling effects, cultural influences, and variability in timing of assessment, which may obscure meaningful differences between different strategies. In contrast, long-term survivorship data remain limited for many alternative philosophies, creating an imbalance between functional and durability evidence.

Finally, the evolving implant designs, polyethylene materials, and advancing technologies mean that the outcomes reported may not fully reflect the effects of different alignment philosophies. Alignment strategies should not be interpreted in isolation from the technological context in which they are applied.

## 6. Conclusions

The philosophy underpinning knee arthroplasties has evolved from a one-size-fits-all to a more nuanced, patient-specific—or, more precisely, collateral-specific—strategy. This shift has been driven largely by advancements in surgical technology, particularly the integration of robotic-assisted platforms, which enable precise execution of personalized alignment and balance goals.

As we better understand which philosophies yield better patient satisfaction, there remains a critical need to ensure that long-term implant survivorship is not compromised in the pursuit of short-term functional gains. Despite the improvements over the years, numerous questions remain unanswered. High-quality, large-scale studies and robust intraoperative data analysis are essential to guide future innovations and refine intraoperative surgical decision-making.

Overall, the strength of evidence across different philosophies remains uneven, with MA supported by the most mature survivorship data, with short- to medium-term data supporting alternative alignments showing favorable PROMs. Understanding these evidence gradients is essential in translating the different alignment strategies into a safe and effective clinical practice.

## Figures and Tables

**Table 1:** Comparison of landmark studies/descriptions, study designs, sample sizes, follow-up periods, outcomes, and levels of evidence across different alignment philosophies.

Alignment Philosophy	Key Landmark/Representative Studies	Study Design(s)	Approx. Sample Size	Follow-up Duration	Primary Outcomes Assessed	Level of Evidence (Overall)
Mechanical alignment (MA)	Insall et al. Bonner et al. National Joint Registries	Retrospective cohorts, registry analyses, comparative studies	Hundreds to >100,000 (registries)	Up to 25 years	Survivorship, aseptic loosening, radiographic alignment	High (long-term survivorship data)
Anatomical alignment (AA)	Hungerford and Krackow; Yim et al.	Conceptual descriptions, RCTs (robotic)	~100	Up to 8 years	PROMs, ROM, joint stability, revision rate	Moderate
Kinematic alignment (KA)	Howell et al.; Elbuluk et al.; Australian/NZ joint registries	RCTs, prospective cohorts, registry data	Hundreds to thousands	Up to 16 years	PROMs, survivorship, satisfaction	Moderate–High
Restricted kinematic alignment (rKA)	Vendittoli et al.; Morcos et al., Abhari et al.	Prospective cohorts, comparative studies	~200–500	Up to 10 years	PROMs, survivorship, satisfaction	Moderate
Inverse kinematic alignment (iKA)	Winnock de Grave et al.; Shichman et al.	Prospective cohorts, comparative studies	~100–300	Short- to mid-term (2–5 years)	PROMs, satisfaction,	Low–Moderate
Adjusted mechanical alignment (aMA)	Vanlommel et al.; Zheng et al.	Retrospective cohorts, comparative studies	~100–200	Up to 10 years	PROMs, satisfaction	Low–Moderate
Functional alignment (FA)	Clark et al.; Young et al.	RCTs, prospective cohorts	~100–700	Short- to mid- term (1–5 years)	PROMs, gap balance, satisfaction	Moderate (evolving)

**Table 2:** Summary of the boundaries recommended by authors initially describing the alignment philosophies. Further details on intra-op modifications, surgical tools, and soft-tissue strategies are included.

Philosophy	rKA	iKA	aMA	FA
Overall limb alignment (HKA)	aHKA within 3 degrees	aHKA between 174 and 183	aHKA within 3 degrees	aHKA between 174 and 183
Tibial component (MPTA)	within 5 degrees	between 84 and 92	perpendicular to the mechanical tibial axis	Between 84 and 93
Femoral component (LDFA)	within 5 degrees	between 84 and 93	between 87 and 93	Between 84 and 93
Soft tissue balance	Restore native joint laxity	Equal gaps in extension and flexion (allow slightly loose lateral flexion)	Flexion gaps 1–2 mm more opening in extension and flexion	Equal gaps in extension and flexion. Allow slightly loose lateral flexion
Priority	Prioritize femoral anatomy	Prioritize tibial anatomy	Depth of femoral resection limited to <3 mm from the joint line	Maintain the femoral position to the joint line, avoid relative varus of the femoral component
Intra-op Modifications	Diseased compartment	Femoral component position	Femoral component position	Initial tibial component positioning followed by the femoral component
Tools	Patient-specific instrumentation (PSI), computer navigation, or robotic-assisted	Initial description with robotic assistance, non-designer authors describe use with conventional instruments	Initial description with robotic assistance, non-designer authors describe use with conventional instruments	Initial description: Robotic-assisted. Expanded to computer navigation by non-designer authors
Soft tissue release strategy	Deep MCL release only expected if MPTA changed from 8 to 5	Medial release in <5% of patients	Not mentioned	0.5% needed MCL fenestration. 3.3% fenestration of LCL ± release of LCL, ITB, or popliteal release

## Data Availability

No new data were created or analyzed in this study. Data sharing is not applicable to this article.

## Abbreviations

The following abbreviations are used in this manuscript:

MA	Mechanical Alignment
TKA	Total Knee Arthroplasty
KA	Kinematic Alignment
rKA	Restricted Kinematic Alignment
iKA	Inverse Kinematic Alignment
aMA	Adjusted Mechanical Alignment
FA	Functional Alignment
LDFA	Lateral Distal Femoral Angle
MPTA	Medial Proximal Tibial Angle
CPAK	Coronal Plane Alignment of the Knee
HKA	Hip–Knee–Ankle
PROM	Patient-Related Outcome Measures
PSG	Patient-Specific Cutting Guide
FJS	Forgotten Joint Score
KOOS QOL	Knee Injury and Osteoarthritis Outcome Score, Quality of Life
AI	Artificial Intelligence
